# How helping others helps us: neural mechanisms linking prosocial behavior to psychological and physical wellbeing

**DOI:** 10.3389/fnhum.2026.1686801

**Published:** 2026-02-23

**Authors:** M. Justin Kim, Sunhae Sul

**Affiliations:** 1 Department of Psychology, Sungkyunkwan University, Seoul, Republic of Korea; 2 Department of Brain Science and Engineering, Sungkyunkwan University, Suwon, Republic of Korea; 3 Center for Neuroscience Imaging Research, Institute for Basic Science, Sungkyunkwan University, Suwon, Republic of Korea; 4 Department of Psychology, Pusan National University, Busan, Republic of Korea

**Keywords:** fMRI, health, prosocial behavior, social brain, wellbeing

## Abstract

Prosocial behavior—voluntary actions intended to benefit others—not only holds moral and social value but also promotes psychological and physical wellbeing through complex neural mechanisms. This Mini Review summarizes current evidence on the health benefits of helping others, including reductions in depression, anxiety, and loneliness, as well as enhancements in positive affect, life satisfaction, and physiological health markers. Then, we provide an overview of the neural systems that support prosociality, highlighting key regions involved in reward processing, empathy, and mentalizing, and how their integration supports flexible, context-sensitive helping behavior. Furthermore, we discuss possible neural pathways linking prosocial actions to stress buffering, mood enhancement, and social connectedness, forming self-reinforcing cycles that sustain wellbeing. We also discuss individual difference factors—including personality traits and early life experiences—that may act to modulate these neural mechanisms and impact prosocial engagement. Understanding how helping others benefits the helper holds promise for advancing population health and fostering resilience across diverse contexts.

## Introduction

Prosocial behavior refers to voluntary actions intended to benefit others, such as helping, sharing, and providing comfort (Batson and Powell, 2003). Helping others is a fundamental human inclination (Piliavin and Charng, 1990) that highlights the human capacity for prosociality, which in turn contributes to a flourishing society (Sober and Wilson, 1998). Of the wide range of its beneficial outcomes—including its direct consequences such as advancing the welfare of others who receive support—accumulating evidence points to an important role of prosocial behavior in promoting psychological and physical health of those who *provide* support (Byrne et al., 2023; Brown and Brown, 2015). Empirical studies show that prosocial behavior is associated with positive mood (Yinon and Landau, 1987), reduced negative effects of stress in everyday life (Raposa et al., 2016), increased longevity (Brown et al., 2003), among other health benefits.

Over the past decades, advances in non-invasive neuroimaging techniques such as functional magnetic resonance imaging (fMRI), along with the rise of social neuroscience as a research field (Ochsner and Lieberman, 2001), have enabled numerous studies to investigate the neural underpinnings of prosocial behavior (Bellucci et al., 2020) and wellbeing (Erickson et al., 2014) in humans. However, few have focused on linking these two psychological constructs (Brown and Brown, 2015; Davidson and McEwen, 2012). This Mini Review aims to provide a summarized account of the current evidence on the neural mechanisms underlying these links. We sought to focus on answering this guiding question: what are the possible neural mechanisms that connects prosocial behavior to health and wellbeing? Specifically, we provide a concise overview of the (1) psychological and physical health correlates of prosocial behavior; (2) neural systems supporting prosocial behavior; (3) neural pathways linking helping behaviors to wellbeing; and (4) individual difference factors that may modulate this connection. By doing so, we sought to unite previously separate neuroimaging literatures on reward, caregiving, and social connection and provide an integrative perspective on this important topic. Given the scope of this Mini Review, we focus on neural systems and pathways most directly relevant to well-being outcomes, rather than providing an exhaustive survey of the prosocial neuroscience literature. Also, because the majority of neuroimaging studies reviewed here are cross-sectional or correlational, directional and causal interpretations should be considered provisional.

### Psychological and physical health correlates of prosocial behavior

A growing body of research indicates that prosocial behavior confers substantial benefits for both psychological and physical health. Engaging in prosocial acts—such as volunteering, supporting others, or performing acts of kindness—is associated with reduced symptoms of depression, anxiety, and loneliness (Aknin et al., 2022; Miles et al., 2022; Raposa et al., 2016). Experimental studies demonstrated that even brief prosocial acts, such as composing a note of appreciation or giving gifts, reliably reduced state loneliness and improve mood (Lanser and Eisenberger, 2023). In terms of enhanced positive affect, life satisfaction, and meaning in life, longitudinal work shows that regular volunteering (e.g., ≥100 h/year) is linked to higher positive affect, optimism, purpose in life, and reduced hopelessness (Kim et al., 2020). Other studies have shown that eudaimonic wellbeing is improved through prosocial acts (Hui et al., 2020; Pearce et al., 2020). Physiologically, prosocial engagement has been linked to reduced stress and enhanced immune function, partly by downregulating pro-inflammatory gene expression (Nelson-Coffey et al., 2017; Regan et al., 2022). However, additional research is required to establish its effects on other physiological systems, including cardiovascular and endocrine function (Trachtenberg, 2024). Finally, longitudinal and meta-analytic studies demonstrated that sustained prosocial engagement predicts greater longevity and reduced mortality risk (Brown et al., 2003; Harris and Thoresen, 2005; Kim et al., 2020; Okun et al., 2013). However, several important limitations should be noted. Effect sizes in the prosociality–wellbeing literature are heterogeneous, suggesting that potential benefits depend on factors such as autonomy, perceived appreciation, and the nature and intensity of helping. Moreover, prosocial behavior does not uniformly confer health benefits; in some contexts, helping can be associated with increased distress and burnout (Lopes Cardozo et al., 2012). Collectively, these findings highlight that prosocial behavior, beyond its moral and social value, may constitute a multifaceted enhancer of wellbeing across body and mind. Consistent with these findings, meta-analytic evidence indicates that prosocial interventions are reliably associated with modest but significant improvements across a range of psychological and health-related outcomes (Byrne et al., 2023).

#### Diverging health impacts of prosocial behavior: self- vs. other-focused motivation

Although prosocial behavior is often associated with positive health-related outcomes, accumulating evidence suggests that not all forms of helping reliably confer benefits to the individual (Oakley, 2013). For example, individual differences in motivational orientation and reward experience (Caprariello and Reis, 2021; Wiwad and Aknin, 2017), sensitivity to others' evaluations (Berman and Silver, 2022), and repeated emotional engagement associated with empathic fatigue (Klimecki and Singer, 2012) have all been identified as conditions under which prosocial behavior may entail psychological costs rather than benefits. Importantly, these effects may vary as a function of motivational processes of prosocial behavior. As proposed by Batson et al. (1983), (2015), prosocial actions driven by self-focused motives such as the alleviation of personal distress may yield qualitatively different emotional and physiological consequences than those motivated by other-focused empathic concern. While empirical evidence remains sparse, converging findings indicate that prosocial actions motivated by self-focused concerns may confer attenuated or negligible benefits to the helper (Caprariello and Reis, 2021; Wiwad and Aknin, 2017). These observations point to motivational orientation—self-focused vs. other-focused—as a central moderator of the relationship between prosocial behavior and wellbeing, which should be considered when discussing the possible neural pathways that connect them. Throughout the remainder of this review, we draw on this distinction based on Batson's framework as a guiding conceptual lens, while acknowledging that current neuroimaging paradigms rarely allow for a clean dissociation between empathic concern and personal distress at the neural level.

#### Diverging health impacts of prosocial behavior: kindness vs. justice

Prosocial acts rooted in kindness are consistently associated with measurable health gains. For instance, Nelson-Coffey et al. (2017) conducted a randomized controlled trial in which performing acts of kindness toward others led to favorable changes in gene expression related to reduced pro-inflammatory and enhanced antiviral immune profiles. Similarly, in experimental stress paradigms, individuals who delivered gift cards to others (vs. to themselves) exhibited faster recovery in cardiovascular markers such as heart rate and diastolic blood pressure following a stressor (Lazar and Eisenberger, 2022). These findings emphasize how affiliative, kindness-driven helping can be associated with lower stress and promote physiological markers of wellbeing.

Intriguingly, prosocial behavior driven by moral or justice concerns may carry distinct emotional and physiological consequences. While less directly studied in health contexts, research suggests that moral-justice motivations, such as enforcing fairness or responding to perceived inequity, can elicit negative emotional responses. For example, greater sensitivity to distributive injustice has been linked to lower wellbeing in children and adolescents (Krettenauer et al., 2019). In adults, higher levels of both victim and altruistic justice sensitivity, reflecting heightened concern for unfair treatment of oneself and others, have been associated with increased negative affect and greater symptoms of generalized anxiety (Zhu et al., 2025). Neuroimaging evidence further indicates that stronger amygdala responses to unfair distributions predict higher depressive symptoms 1 year later (Tanaka et al., 2017). A recent study found that individuals high in justice sensitivity were more likely to make costly prosocial decisions when facing advantageous inequity, a pattern mediated by heightened feelings of guilt rather than pure empathic concern (Zhu and Liu, 2024). Importantly, these associations are likely to vary depending on dispositional and contextual factors such as empathy, socioeconomic status, and the form of justice-related action (Lee et al., 2025).

Taken together, although both kindness- and justice-based prosocial behaviors serve important societal functions, their psychological and physiological health consequences are likely to differ. Kindness-driven helping tends to be affiliative and soothing, fostering wellbeing, whereas prosocial actions anchored in moral obligation or justice may invoke negative affect, with potential health costs. Continued empirical investigation is essential to delineate the distinct neural pathways by which various types of prosocial behavior impact wellbeing.

### Neural systems for prosocial behavior

Encompassing behaviors such as helping, sharing, and comforting, prosocial behavior is thought to depend on an integrated network of neural systems that subserve social cognition, affective processing, and reward valuation. Converging evidence from neuroimaging, brain stimulation, and lesion studies highlights three broad networks. Importantly, these neural systems are not expected to map uniquely onto empathic concern or personal distress; rather, their relative engagement is likely to vary depending on motivational orientation, contextual demands, and task design.

#### Default mode network

The default mode network (DMN) is a large-scale brain network encompassing the medial prefrontal cortex (mPFC), posterior cingulate cortex (PCC), and the precuneus, among other midline and lateral parietal regions (Buckner and DiNicola, 2019). Traditionally associated with internally directed thought such as autobiographical memory retrieval, self-referential processing, and future simulation (Buckner and Carroll, 2007), the DMN is increasingly recognized as a key neural substrate for prosocial behavior. Functional MRI studies have shown that DMN regions are engaged during mentalizing and perspective-taking tasks, processes essential for understanding others' intentions and emotions (Mars et al., 2012). Importantly, the DMN is also implicated in self-referential and moral cognition, with mPFC and PCC activation linked to evaluating one's own moral values, integrating personal identity with ethical norms, and making decisions that balance self-interest with the welfare of others (Greene et al., 2001; Reniers et al., 2012). This capacity to merge self-related and other-related representations, which is known to be represented in the mPFC (Wittmann et al., 2016) may enable individuals to perceive helping behaviors as personally meaningful, thereby increasing the motivation to act prosocially (Sul et al., 2015). We note that, likely owing to its known function in self-related processing, DMN involvement in prosocial behavior is most often observed in contexts that require self-other reflection, moral evaluation, or future-oriented simulation, rather than across all forms of helping. Together, these findings position the DMN not only as a social cognition hub, but also as a neural bridge between self-awareness, moral reasoning, and sustained prosocial motivation. Within the Batson framework, DMN engagement may be more pronounced during forms of helping that involve perspective-taking, moral reflection, or identity-relevant meaning—features more closely aligned with other-focused empathic concern—though direct empirical tests of this distinction remain limited.

#### Empathy and mentalizing networks

Empathy and mentalizing networks constitute fundamental neural substrates underpinning prosocial behavior by enabling individuals to understand and resonate with the internal states of others. Empathy, often conceptualized as the capacity to share or vicariously experience another's emotional state, primarily engages the anterior insula (AI) and the anterior cingulate cortex (ACC), regions implicated in affective processing and the representation of bodily states. Neuroimaging studies have consistently demonstrated that these areas are activated both when individuals experience pain themselves and when they observe others in pain, highlighting a shared affective resonance mechanism critical for empathic concern (Bernhardt and Singer, 2012). Critically, heightened activation in affect-sharing regions such as the AI and ACC may reflect either empathic concern or aversive personal distress, depending on regulatory context and motivational orientation, underscoring the importance of distinguishing these states at the psychological level even when neural activations overlap. Mentalizing, or theory of mind, refers to the cognitive capacity to infer others' beliefs, intentions, and perspectives, recruiting a distinct but interconnected network centered on the mPFC, temporoparietal junction (TPJ), and posterior superior temporal sulcus (pSTS) (Gallagher and Frith, 2003). These regions facilitate perspective-taking and the attribution of mental states, processes essential for understanding social cues and motivating context-appropriate prosocial responses. For example, activation of the mPFC and pSTS has been consistently observed when individuals engage in tasks involving charitable giving or cooperative decision-making (Rilling et al., 2004; Waytz et al., 2012). Importantly, the integration of empathy and mentalizing networks enables nuanced social cognition, whereby affective resonance is modulated by cognitive appraisal, allowing individuals to flexibly engage in helping behaviors that consider both emotional and situational factors (Bellucci et al., 2020; Decety and Jackson, 2004; Hein and Singer, 2008). Disruptions in these neural circuits have been linked to impairments in social functioning and reduced prosociality in clinical populations (Arioli et al., 2021). Collectively, the coordinated activity within and between empathy and mentalizing networks forms the neural foundation for prosocial behavior, driving motivation to alleviate others' distress and engage in cooperative social interactions.

#### Reward and motivation systems

Prosocial acts frequently activate the brain's reward circuitry, including the ventral striatum, ventromedial prefrontal cortex (vmPFC), and midbrain dopaminergic regions such as the ventral tegmental area (VTA). Of note, the ventral striatum, a subcortical structure that encompasses the nucleus accumbens, is well recognized for its involvement in positive affect, reward processing, and valuation, integrating both primary and social rewards (Berridge and Kringelbach, 2015; Wang et al., 2016). Functional neuroimaging research consistently demonstrates that monetary and charitable donations elicit ventral striatal and vmPFC responses comparable to those evoked by primary and self-relevant rewards, consistent with the notion that helping can be intrinsically rewarding (Moll et al., 2006; Harbaugh et al., 2007). The VTA, a major source of dopaminergic projections, may support prosocial behaviors by signaling reward prediction errors (Schultz et al., 1997), thereby strengthening the association between helping others and internal reward states (Xie et al., 2023). Moreover, orbitofrontal and ventromedial prefrontal cortices integrate reward-related signals with contextual and social information (Ruff and Fehr, 2014), allowing individuals to evaluate the relative benefits of self- vs. other-oriented actions. Importantly, the same neural systems implicated in the hedonic experience of receiving rewards appear to be co-opted in the context of giving, supporting the “warm glow” hypothesis of altruism (Andreoni, 1990). Experimental work further demonstrates that social context modulates these reward signals—for example, the presence of observers amplifies ventral striatum activation during giving, linking reputational, and prosocial motives to mesolimbic valuation (Izuma et al., 2010). Intriguingly, these same reward circuits have important implications for beneficial health outcomes, providing a possible link between prosocial behavior and wellbeing (Dutcher, 2023; Telzer, 2016). Together, these findings suggest that prosociality is not merely a moral obligation but is intrinsically rewarding, underpinned by neurobiological systems that motivate and sustain such behavior.

### Neural pathways linking helping to wellbeing

Engaging in prosocial behavior appears to be associated with psychological and physical wellbeing through interconnected neural mechanisms that reinforce and sustain helping over time. Based on accumulating neuroimaging evidence on prosociality and wellbeing, we propose four pathways through which prosocial behavior leads to improved psychological and physical health: (1) positive feedback loop; (2) stress buffering; (3) reduced self-focus and rumination; and (4) enhanced social connectedness. These pathways should be understood as integrative, higher-level models rather than fully specified mechanisms, reflecting synthesis across heterogeneous literatures that differ in task design, temporal scale, and outcome measures.

First, helping others can initiate a positive feedback loop in which activation of mesolimbic reward circuitry—including the ventral striatum, vmPFC, and VTA—elicits positive affect, thereby enhancing mood and increasing the likelihood of future helping behaviors. Neuroimaging evidence suggests that the ventral striatum in particular mediates motivation and reward during prosocial decisions, creating a self-reinforcing loop that sustains wellbeing over time (Harbaugh et al., 2007; Moll et al., 2006). This dopaminergically mediated reward response is most likely to operate under conditions in which helping is voluntary, positively valued, and perceived as effective, and concurrent evidence for downstream health benefits remains largely indirect.

Second, helping may buffer stress by recruiting caregiving-related neural circuits, such as the septal area and hypothalamic-pituitary-adrenal (HPA) axis-modulating oxytocinergic pathways, which have been implicated in attenuating physiological stress reactivity and promoting calm, affiliative states (Inagaki, 2018). One possibility is that the neural regions associated with caregiving, such as the ventral striatum and septal area, may play dual roles by reinforcing support-giving behaviors and attenuating stress responses, including those in the amygdala and other threat-related circuits (Brown and Brown, 2015; Inagaki, 2018). Concurrently, oxytocinergic pathways appears to supplement this effect, such that oxytocin release from the hypothalamus has been shown to dampen HPA-axis activation, lower cortisol levels, and reduce amygdala reactivity to social threats and stressors (Eisenberger, 2013; Smith and Wang, 2014). This notion is further supported at the behavioral genetics level, where oxytocin receptor genes and prosocial behavior may interact to reduce stress and improve physical health (Poulin and Holman, 2013). It appears that empirical support for this pathway is strongest in caregiving and support-giving contexts, whereas its generalizability to other forms of prosocial behavior remains an open question.

Third, by directing attention outward toward the needs of others, prosocial engagement may reduce maladaptive self-focus and rumination, processes often linked to hyperactivity (Freton et al., 2014) and abnormal connectivity (Greicius et al., 2007) of the DMN in depression (Kaiser et al., 2015). While direct evidence tying helping to DMN modulation is still emerging, intervention studies (e.g., mindfulness training) suggest that fostering external focus and engagement can enhance network switching, reducing DMN dominance (Bremer et al., 2022). We speculate that regulated DMN activity and connectivity during helping-related tasks may therefore contribute to improved mood regulation and cognitive flexibility. At present, this pathway remains primarily theoretical, as few neuroimaging studies have directly examined DMN dynamics during helping behavior itself.

Finally, neural rewards associated with prosocial behavior—particularly within ventral striatal and orbitofrontal regions—may serve to strengthen social bonds, improving perceived social connectedness and belonging. fMRI studies demonstrated that engaging in reciprocal cooperation during interpersonal paradigms, such as the Prisoner's Dilemma, elicits robust activation within the brain's reward circuitry (Decety et al., 2004; Rilling et al., 2002). This reinforcement of affiliative ties not only fosters further prosociality but also supports resilience and wellbeing through the maintenance of meaningful social relationships. We propose that prosocial individuals are not only more likely to receive social support (Wilson et al., 2009), but engaging in prosocial behavior also enhances the helper's psychological and physical wellbeing by fostering a greater sense of social connectedness (Kim and Sul, 2023; Morelli et al., 2015). This pathway is best supported in paradigms involving reciprocal interaction and repeated social exchange, and less is known about its operation in one-shot or anonymous prosocial acts.

It is worth noting that while neuroimaging evidence for psychological wellbeing is more readily available than its physical counterpart. That said, engagement of reward, caregiving, and default mode network circuits during prosocial behavior has been linked to downstream psychophysiological pathways—such as oxytocinergic signaling, HPA axis regulation, and autonomic control—that are known to influence inflammation, cardiovascular function, and broader immune health over time (Brown and Brown, 2015; Marsland et al., 2017; Telzer, 2016). To summarize, these pathways suggest that the brain mechanisms engaged during helping create a self-reinforcing cycle that links prosocial acts to sustained psychological and physiological benefits.

### Individual difference factors to consider

Individual differences that shape the prosocial–wellbeing link can be broadly grouped into developmental and dispositional factors. Neural responses to prosocial behavior vary across the lifespan, reflecting developmental changes in social and motivational brain circuits, including the vmPFC (Westhoff et al., 2021), pSTS, temporal pole, and inferior frontal gyrus (Do et al., 2019) during prosocial decision-making. Personality and early life experience additionally modulate neural responses to prosocial acts, including greater TPJ activation associated with enhanced mentalizing engagement (Haas et al., 2015) and reward-related neural functioning associated with early caregiving experience (Morgan et al., 2014; Hanson et al., 2016). In sum, these findings underscore that both developmental stage and individual differences—rooted in personality traits and early environmental influences—may shape the neural architecture underlying prosocial behavior. Individual differences in personality, developmental stage, and early-life environment would influence both the likelihood of engaging in prosocial behavior and its downstream neural and physiological effects. Understanding how these individual difference factors influence neural processes is essential for clarifying the mechanisms linking prosocial behavior to health outcomes, as they may hold the key to developing a comprehensive explanatory framework.

## Conclusions

By prioritizing conceptual integration over exhaustive coverage, our goal was to clarify key neural pathways linking prosocial behavior to well-being and to highlight testable directions for future empirical work. Helping others is not only morally and socially valuable, but also intrinsically rewarding at the neurobiological level, engaging reward-related, stress-buffering, and social-bonding circuits. Converging evidence from social neuroscience reveals multiple, interacting neural pathways that may account for the observed links between prosocial behavior and both mental and physical wellbeing. This growing understanding highlights the potential of emphasizing prosociality as a strategy to enhance population health and resilience. However, important gaps remain. Longitudinal and causal studies are needed to clarify directionality and causality—whether helping behavior induces lasting neural changes or whether pre-existing neural traits predispose individuals toward prosociality. Most of the currently available neuroimaging evidence is cross-sectional, and as such the directionality between prosociality and brain function is unclear. Physical health pathways, including possible neuroimmune and neuroendocrine mechanisms, remain underexplored and more empirical studies are needed. Given the societal importance of prosocial behavior in heterogeneous societies (Baldassarri and Abascal, 2020), future work must also address diversity and generalizability, examining how these mechanisms operate across cultures, socioeconomic backgrounds, and health conditions. Finally, translating these insights into neuroscience-informed interventions and informing existing prosocial training programs (Baumsteiger, 2019; Byrne et al., 2023) could open new clinical avenues to support psychological wellbeing, strengthen social connections, and promote healthier, more resilient communities.
